# Enhancing addition fact fluency in children with mild intellectual disabilities: simultaneous prompting with performance feedback

**DOI:** 10.1186/s40359-025-03311-w

**Published:** 2025-08-17

**Authors:** Nesrin Sönmez, Serpil Alptekin

**Affiliations:** 1 Special Education Department, Akdeniz University, Antalya, Turkey; 2 Special Education Department, Ondokuz Mayis University, Samsun, Turkey; 3 Akdeniz University Educational Faculty Dumlupınar Bulvarı, 07058, 4400/4619 Kampüs, Antalya, Turkey

**Keywords:** Fluency, Intellectual disabilities, Mathematics, Performance feedback, Simultaneous prompting

## Abstract

**Background:**

The development of mathematical skills in students with intellectual disabilities can facilitate greater independence in everyday life. The ability to perform basic addition facts (BAFs) with fluency can facilitate the learning process of mathematics and the ability to perform quickly and correctly on a subject. This study examined the impact of simultaneous prompting with performance feedback (SP-PF) on fluency in BAFs.

**Methods:**

Three high school students with mild intellectual disabilities participated in the study, which employed a multiple-probe, across-participants design.

**Results:**

The results demonstrated that the SP-PF effectively enhanced the participants’ fluency in BAFs to the criterion level. All participants demonstrated maintenance of their fluency levels for at least 15 days following the instruction. On day 45, two students exhibited fluency levels that were very close to the criterion. Social validity data indicated that the participants were satisfied with the study procedures and their learning outcomes. Furthermore, social comparison demonstrated significant effects.

**Conclusions:**

This study contributes to the literature on the effectiveness of the SP-PF intervention on fluency in BAFs in students with special needs. It shows that participants demonstrated the ability to answer facts without using their fingers.

## Background

Fluency in basic facts is defined as the ability to perform mathematical facts either orally or in writing with speed and accuracy [1]. An individual with enhanced processing fluency can concentrate solely on the specific skill being learnt, as they are not preoccupied with the need to complete the task in a timely manner [2]. This allows them to ignore distracting elements and focus on the new task for a longer duration [3]. Consequently, the student’s motivation and effort increase [4]. Students who lack proficiency in fundamental concepts may resort to time-consuming strategies such as finger counting, which impair efficiency and, consequently, the motivation necessary for success [5]. Given that these factors affect all learners, rather than just those with disabilities, it is becoming increasingly clear that improving fluency in facts is an important aspect of mathematics education.

Kearns et al. [6] found that all grades’ worth of pupils with intellectual disabilities exhibited extremely poor arithmetic proficiency. It is possible to achieve the goals if well-planned and effective teaching elements are provided. Systematic instruction is a crucial element in the education of students who exhibit poor mathematical performance [7, 8]. Several effective instructional strategies for teaching mathematics to children with intellectual disabilities include prompting strategies [9]. One such strategy is simultaneous prompting (SP), which minimises the possibility of mistakes being made by students [10].

SP is a prompting strategy used in teaching discrete and chained skills in various areas [10, 11] and considered an evidence-based practice for special learners [10]. The SP instructional trial involves teachers delivering controlling prompts to students after presenting target stimuli [12]. Instruction continues in this manner, followed by training trials, until the criterion is met during daily probe trials. It is stated that the SP procedure can be described as a promising application in teaching mathematics content to disabled students [13]. SP facilitates students’ development of independence and fluency in mathematics by providing the appropriate support without exceeding the scope of each student’s needs [14]. Although numerous studies have demonstrated the efficacy of SP in enhancing teaching skills, it is acknowledged that further research is required to elucidate the impact of SP procedures in academic domains [10].

The impact of the SP procedure has been demonstrated in the acquisition of discrete mathematical skills, including subitizing [15], pointing to numbers [16], multiplication facts [17], and identifying geometric figures [18]. Morse [13] reported that three of the eleven studies with discrete tasks considered the acquisition of the basic addition facts (BAFs) as one of the dependent variables in the interventions. Despite the evidence base for SP procedures in teaching individuals with intellectual disabilities, it is surprising that no study has investigated the impact of SP procedures on improving fluency in BAFs. However, only two studies [17, 19] have demonstrated its efficacy in facilitating fluency in multiplication facts. The procedures employed in fluency studies frequently encompass a combination of instructional components. For instance, Sonmez and Alptekin [19] used the SP instructional package in conjunction with a systematic review and corrective feedback.

Feedback is a crucial communication tool that provides specific details or critiques about someone’s behaviour. In education, one of the most valuable tools for giving students feedback is often regarded as an essential element of effective teaching [20, 21]. Browder et al. [22] found that systematic instruction, including the use of a prompt fading method with feedback, is an evidence-based procedure for teaching mathematics. Schnepel and Aunio [9] similarly found in their systematic review study that feedback was a component of all the mathematics interventions for primary school students with intellectual disabilities. The current study aimed to investigate the effect of using performance feedback with SP.

Performance feedback (PF) is given to students about their performance in a direct, precise manner regarding the form, context, correctness, or frequency of what they do [21], and can be verbal, written, or visual. Graphs that enable data visualisation are frequently employed tools. Teachers may be able to more accurately and frequently assess patterns in student performance if data is presented as a graph. Furthermore, graphs may also facilitate the delivery of feedback to students on a more frequent basis [23]. Gersten et al. [20] found their meta-analysis study that providing students with information on their progress in graphic form was statistically significant. In contrast, one study investigated the difference in student performance between intervention packages with and without PF and found that each intervention had a similar effect [24]. There is limited evidence regarding PF, as evidenced by the disparate findings of studies examining the combined impact of PF added to various approaches on various target behaviours. However, it is evident that the forms of giving PF used in these studies are common, and PF is given to students graphically.

In the studies of fluency in mathematics, PF is included as an element of the self-management intervention package, with participants monitoring their progress using self-graphs. These studies have demonstrated that PF (in the form of self-graphing) and goal setting can improve students’ fluency [25, 26]. It has been demonstrated in fluency studies that the incorporation of PF into the intervention leads to enhanced performance [3, 27]. To date, no study has examined the effects of SP with PF on basic fact fluency or on other areas of mathematics for students requiring more systematic teaching. In prompting procedures research, it was observed that the instructional procedure combined with different feedback strategies, such as corrective feedback [19] or instructional feedback [28, 29], was effective. It was also observed that fluency studies with SP did not examine fluency in BAFs as a target skill. Therefore, it is hoped that this study will address this gap in the field by evaluating the effect of SP in the mathematics domain as a target skill and by studying the impact of the combined use of PF given by the implementer. The primary objective of this study is to ascertain the efficacy of simultaneous prompting with performance feedback (SP-PF) in enhancing fluency in BAFs for students with mild intellectual disabilities. The study specifically aims to address the following questions:

1. Does the SP-PF facilitate the acquisition of fluency in BAFs by students with mild intellectual disabilities?
2. Does the SP-PF facilitate the maintenance of the fluency levels of students with mild intellectual disabilities?
3. Are students with mild intellectual disabilities able to generalize the level of fluency acquired to different BAFs?
4. Will the participants’ fluency levels reach the level of their typically developing peers?
5. To what extent are the results of the research socially significant?

## Materials and methods

### Participants

The participants in the study were three students with mild intellectual disabilities who met the research participation criteria. To be eligible for participation in the study, students were required to demonstrate a 90% accuracy rate in solving BAFs, albeit at a slower pace (e.g., by counting fingers). Additionally, they were required to have no other disabilities besides intellectual disabilities and provide written informed consent from their parent, and maintain regular school attendance. Prior to commencing the study, the researchers obtained institutional ethical approval. The selection of participants was based on the completion of a Student Information Form, a Teacher Interview Form, and the administration of separately developed checklists for basic facts (addition, subtraction, multiplication, division) created by the researchers. The demographic information of each participant (including medical and educational diagnoses, age, etc.) was obtained from the school administration.

To identify participants who met the research criteria, preliminary interviews were conducted with teachers and students. Students who could perform BAFs (with numbers 1–9) but lacked sufficient speed were identified through one-on-one sessions. Of the 12 students initially reported, four were excluded for not meeting the 90% accuracy criterion, and three due to additional disabilities. The parents of the remaining five eligible students were interviewed individually, during which ethics committee permissions were shared, and consent was obtained. Among the five students who met all the participation criteria, three were included as participants in the main study, as detailed in Table 1. Of the remaining two students, one was assigned as a substitute participant to address any unforeseen circumstances, and the other was designated as a pilot participant to test and refine the study’s procedures (see the General Procedures). The characteristics of the participants are presented in Table 1.

**Table 1:** Descriptions of the participants

Students	Age	Grade	Gender	Diagnosis	Medication	Criteria of Fluency
Doruk	15	10	Male	Mild intellectual disabilities	No	15
Selin	17	12	Female	Mild intellectual disabilities	No	17
Zehra	17	12	Female	Mild intellectual disabilities	No	16

Doruk has literacy skills and can answer questions about the text. In mathematics, he can add with his fingers, count by heart up to 500, count by skipping, and count backwards from 20 by 1. He recognises three-digit numbers and can write two-digit numbers. Although he has good fine and gross motor skills, he needs constant reminders to return to work when doing tasks that require attention. He can follow the curriculum in the sheltered workshop at school. Selin is literate and can answer questions about the text she reads. In mathematics, she can do finger addition and count by heart up to one hundred, and backwards from 20 by 1. She can recognise and write two-digit numbers. She receives vocational training in the school’s hairdressing workshop and can follow the curriculum. In general, she is a student who follows the rules. Zehra has literacy skills and can answer questions about the text. In mathematics, she can add with her fingers, count by rote up to 100, and backwards from 5 to 1. She can identify and write two-digit numbers. She receives vocational training in the hairdressing workshop at school but needs help with fine motor skills. She often speaks out of context in class. She may behave in a defiant manner towards authority.

### Implementer and observer

The first author served as the implementer in the study, and the second author was responsible for the collection of reliability data for all sessions as a secondary observer. Both researchers are specialists in the field of special education, holding positions as academics at two public universities and having obtained doctoral degrees.

### Setting and materials

The study was conducted at a public special education vocational school situated in the centre of Antalya city. The school for students with mild intellectual disabilities (including Down syndrome and autism) offers middle and high school programs, each lasting four years. Its primary goal is to develop vocational skills, supported by a specialized curriculum. Vocational workshops include kitchen, hairdressing, and hotel services, alongside academic subjects such as mathematics.

All sessions were conducted in a self-contained room within the school. The room dimensions were 5 m × 3 m, and it was furnished with two tables (one large and one small) and two chairs. The implementer and the student were positioned face-to-face. To ensure the confidentiality of the participants, the tripod and video camera were positioned on the side of the study table, with the student’s face obscured by the angle of the camera. This was done with the consent of the parents. The data recording forms (in all sessions) and PF graph (in daily probes only) were placed on the table with the hand used by the implementer on the side. A worksheet, a pencil, and an eraser were made available to the participant in advance. The reinforcers for the participants, which included reading books, listening to music, eating chocolate and pastries, and drinking tea, were kept ready on a small table in the far corner of the room.

### Dependent and independent variables

The dependent variable of the study is the participants’ level of fluency in BAFs. The data demonstrates the participants’ level of fluency in BAFs, as evidenced by their ability to solve the BAFs items within one minute. The BAFs includes facts that can be performed with numbers less than 10 [8]. The independent variable in this research is the intervention of the SP-PF.

### Experimental design

A multiple-probe, across-participants design was employed to assess the efficacy of the SP-PF in enhancing fluency in BAFs among students with mild intellectual disabilities. In this model, the same intervention is evaluated by applying the same target behaviour to different participants under equivalent conditions, and the effectiveness of the application is determined [30]. This approach focuses on demonstrating the functional relationship between intervention and outcome within individual participants, which is particularly relevant for the target population [31]. Such populations often require working with a limited sample, both due to the difficulty of finding participants who fulfil certain inclusion criteria and the necessity of intensive individualised interventions. All procedures in this study were approved by the Akdeniz University Rectorate Social and Human Sciences Scientific Research and Publication Ethics Committee (approval no. 265, 21.07.2022). The procedures followed were in accordance with the Helsinki Declaration. Written informed consent to participate was obtained from the parents.

### General procedures

Following the selection of participants, their teachers were contacted to provide a brief explanation of the study’s procedures. It was emphasised that participants should refrain from practicing addition facts until the study was completed. A pilot study was conducted to determine the duration of each step in the study (data collection and intervention) and to assess the suitability of the data collection tools, instructions, environment, and materials. To this end, pilot study sessions were conducted with the student selected as a pilot participant. While the interventions were held during the intervention with the main study participants, the teacher was warned not to conduct fluency in BAFs with the substitute participant who was selected to work in case of a possible loss of participants.

Interventions were held during the participants’ class hours on weekdays and immediately following the daily probe sessions. Two teaching sessions were held per day. Two trials were conducted in each teaching session. There is a 1–2-minute break between trials. Each trial lasted approximately 8 min, and each teaching session lasted 20 min in total. The daily probe data was collected at the beginning of the first instructional session each day (from after the first day of the intervention). Daily probe sessions were conducted in 1-minute sessions. Intervention sessions continued in three consecutive probe sessions until the fluency criterion target determined for the participant was reached. At least one lesson hour (40 min) was waited between sessions. If daily probe sessions were required with other participants in the same day, these probes were held during this break.

#### Determining of participants’ fluency criterion

The criterion for written responses to basic facts was determined by calculating two-thirds of the student’s number writing performance, as outlined by Stein et al. [8]. In the one-on-one session with each participant, the implementer set a timer for one minute and requested that the participants write the numbers spoken (from 1 to 9) as quickly as possible, repeating the same numbers until the time ran out. At the conclusion of the designated period, the participant was terminated, and two-thirds of the result was calculated by tallying the numbers they had written. The fluency criterion was determined to be 15 for Doruk, 17 for Selin, and 16 for Zehra (see Table 1).

### Dependent measures

In this study, data on the effectiveness, generalization, social validity, and maintenance of the intervention were gathered and examined. To collect data on effectiveness, the “Basic Addition Data Chart” was employed during the baseline, intervention, daily probes, generalization, and maintenance sessions of the study. This was used to record the participants’ fluency performance in BAFs. The worksheets, which were based on the participants’ number writing performances, were employed in the data collection and intervention sessions of the study. Given that the participants’ fluency criteria fell below 20 facts per minute, 20 facts were written on the worksheets. The worksheet, which was prepared by rearranging the positions of the 20 BAFs items from the baseline, daily probe, and maintenance sessions (without altering the locations of the addends), was utilized in the intervention sessions. In the generalization sessions, worksheets were employed that included the facts identical to those utilized in the probe sessions. However, both the facts and the addends were replaced. The data collection tools were developed by the researchers.

#### Social validity

Social comparison and subjective evaluation were used to assess the importance of the research, the meaningfulness of the findings, and the acceptability and relevance of the instructions. Social comparisons were conducted between two different groups. The first group consisted of primary school students from schools located in two districts with differing socioeconomic statuses (SES). It was collected from a total of 193 typically developing students: 50 in the first grade, 41 in the second grade, 53 in the third grade, and 49 in the fourth grade. The second group was selected from the participants’ peers attending the same school. To this end, eight students who demonstrated proficiency in fluent addition were identified through interviews with their teachers from grades 9, 10, 11, and 12. In this second group, addition fluency was assessed in two students from grade 9, three students from grade 10, two students from grade 11, and one student from grade 12. For collecting the social comparison data, the “Basic Addition Fluency Test” was administered for both groups. While developing this test, a worksheet containing different BAFs was used in addition to the BAFs used in the teaching set.

Regarding the subjective evaluation data, interviews with the participants were conducted using both Likert-type questions and open-ended forms. To collect the subjective evaluation data, an interview form was developed by the researchers. The purpose of the form is to evaluate the strengths and weaknesses of the SP-PF procedure and its impact on participants based on their feedback. The form includes a total of eight questions: six closed-ended and two open-ended. The questions in the form were adapted from social validity forms used in previous single-subject studies.

## Experimental procedures

### Baseline

Before the SP-PF teaching sessions, baseline data were collected for each participant until stable data were obtained for at least three consecutive sessions. The environment and materials were briefly introduced, and after a brief conversation with the participant, a sentence was directed to start the study and attract the participant’s attention (e.g., “Are you ready to work with me?“). After the participant’s attention was captured, this behaviour was verbally praised (e.g., “Great, I see you are ready!”). Then, it was explained to the participant what she/he would do in the study: " With my command to start, you will start solving the facts on this paper. When time is up, I will say stop, and at the same time, you must put down your pen immediately. If I have not said stop when the facts are completed, it will be blank. You can look again at what you left behind.” While setting the stopwatch for 1 min, the practitioner gave the command “Ready, start” at the same time. When one minute was completed, the implementer said “stop” to the participant. The implementer ended the session by thanking her/him for her/his participation. To ensure that there was no slight change in the participant’s performance, no prompts or reinforcements were provided to the participant. The implementer counted the collection facts that the participant answered correctly within 1 min and wrote this number on the data recording form.

### Intervention

After preparing the setting and materials for instructing, the participant was taken to the study room. After a short conversation, the participant was informed about the study to be conducted and was reminded of the rules to be followed in the study. “Today we will work with you on the facts in this worksheet. I will read the facts and tell you the results. You should listen to me carefully. Then I will ask you for the answer to the fact.” The implementer then showed or explained to the participant the reward he or she would receive at the end of the study and started the study by giving an attention sign. The implementer read the first fact on the worksheet and said the answer. “Four plus five equals nine. " Now tell me, how much is four plus five?“. If the participant answered correctly, the implementer reinforced her/him by saying, “Well done; four plus five equals nine, you said it right.” If the participant gave an incorrect, indecisive response or did not respond at all, the implementer gave corrective feedback by repeating the correct answer as “four plus five equals nine.” When the first trial of the teaching session was completed in this way, a short break (1–2 minutes) was given to the teaching. After the break, the second teaching trial was started, and the processes in the first trial were repeated. After two trials, the teaching session was completed, and the study ended with the reward given to the participant.

### Daily probes

Daily probe sessions were administered immediately before the teaching sessions to evaluate the level of fluency in BAFs of the participants. The fluency performance obtained because of the probe, which was conducted by following the same processes applied in the baseline sessions, was recorded on the line graph. If there was an increase compared to the previous probe, this situation was reinforced (e.g., look, you made ten correct facts; yesterday it was nine. Your correct answers are increasing! ). Otherwise, the student was told that she/he could increase the number of correct answers if she/he worked more carefully tomorrow.

### Generalization

Following the achievement, the criterion of fluency in BAFs by the participants, generalization measurements were conducted using a generalization set that was created by rearranging the locations of the addends and facts. Using the same protocol as baseline measures, generalization sessions were held before and after the intervention. Participants did not receive prompts, feedback, or reinforcement for their responses in the generalization sessions.

### Maintenance

The maintenance sessions were conducted 7, 15, and 45 days following each participant’s completion of the fluency criterion. The third maintenance session for Zehra was unable to take place because of the school closure. The baseline and daily probe methods were followed throughout maintenance sessions.

### Interobserver agreement

Interobserver agreement (IOA) of the dependent variable measurement was obtained across at least 25% of sessions for the baseline, daily probe, generalization, and maintenance. The implementer served as the major observer, and the second author served as the secondary observer. Both are experts in single-subject study designs and direct observations. All sessions were recorded, and the sessions for IOA were chosen randomly. For the analysis of IOA data, the formula “[agreement / (agreement + disagreement) × 100]” was employed [31]. IOA percentages were 100% in the baseline, daily probe, generalization, and maintenance sessions for all participants.

### Procedural fidelity

Procedural fidelity was assessed for a minimum of 20% of the intervention sessions, which were selected randomly for all participants using the “SP-PF Procedural Fidelity Checklist” developed by the second author and used to determine whether the implementer applied the SP-PF application steps correctly. The second author took part in observing the implementer behaviours. The procedural fidelity was calculated as the number of implementer behaviours observed divided by the number of implementer behaviours planned, multiplied by one hundred, and was found to be 100% for all observed sessions of each participant.

### Data analysis

Data obtained during all intervention sessions were presented graphically, and the graphs were visually analysed in this study. Each participants’ fluency performance on the BAFs in each session of the study was displayed on the graphic to evaluate the effectiveness of SP-PF for each participant. Furthermore, the data were entered into the Tau-U calculation tool [32] to get the effect size scores for each participant. Values between 0 and 1 resulting from the calculations made with Tau-U were interpreted as 0-0.65 as a low-level effect, 0.66-0.92 as a medium-level effect, and 0.93 − 1.0 as a high-level effect [33]. Social validity data were analysed in two ways: qualitatively and graphically. For the first way, the data gathered from the interviews with the participants was analysed through descriptive analysis. For the second way, the social comparison data was shown with a column chart and analysed visually.

## Results

### Effectiveness

**Fig. 1:**
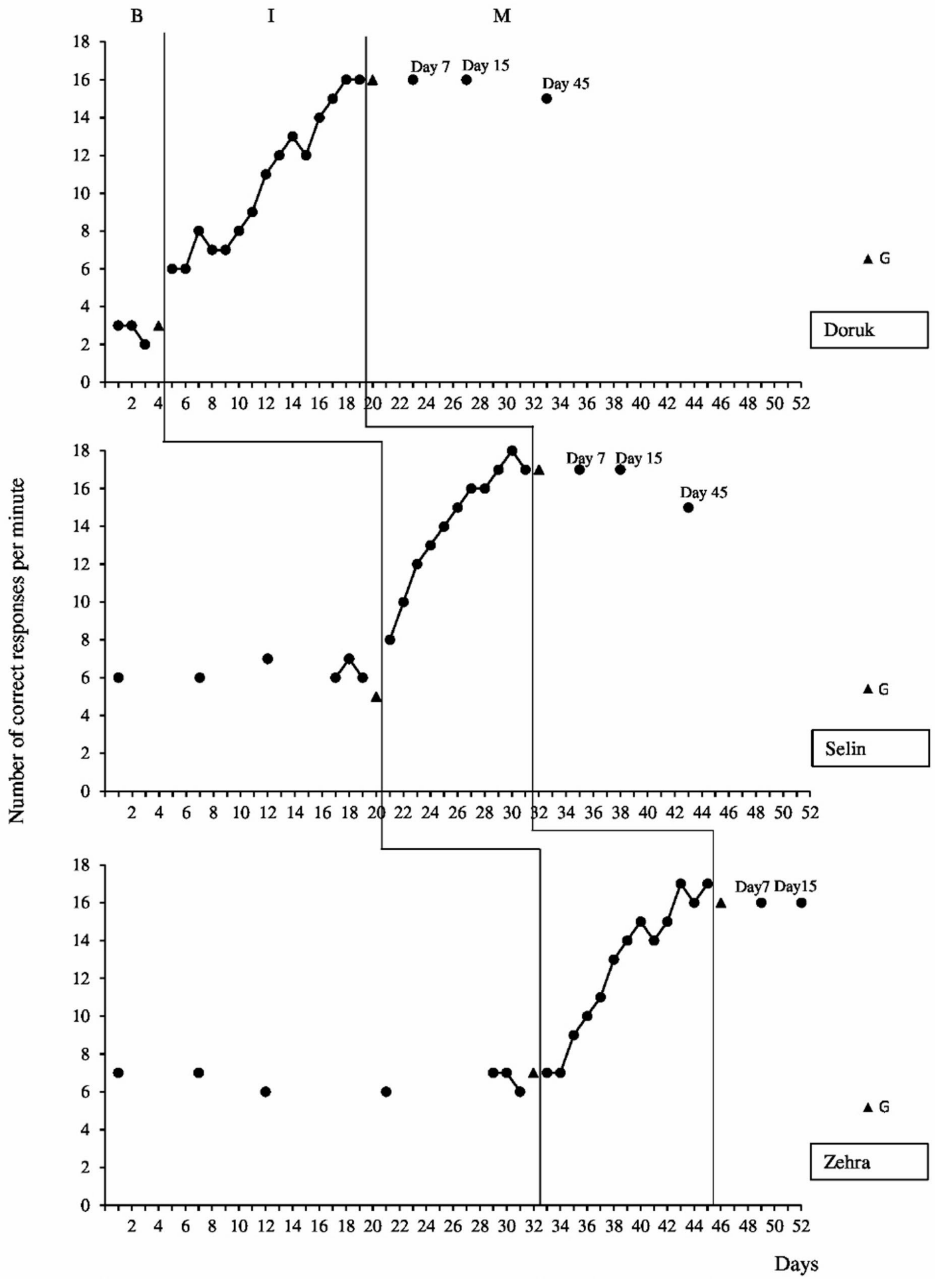
The level of addition fact fluency for Doruk, Selin, and Zehra during the baseline (B), intervention (I), generalization (G), and maintenance (M) sessions

Figure 1 shows Doruk, Selin, and Zehra’s fluency levels in the baseline, intervention generalization, and maintenance sessions. There was a significant increase in all participants fluency in BAFs performance after the intervention started. On the 13th day of the SP-PF sessions with Doruk, it was observed that he performed at the criterion level. There was a significant increase in Doruk’s fluency performance in the intervention phase (average 15.6) compared to the baseline level (average 2.6). Selin increased the level of fluency from the beginning of the intervention without any tendency to fall. On the 9th day of the SP-PF sessions with Selin, it was observed that she performed at criterion level. There was a significant increase in Selin’s fluency performance in the intervention phase (average 17.3) compared to the baseline level (average 6.3). Zehra’s fluency performance started to increase from the third day of the intervention and increased until the ninth day. Although there were small ups and downs in the following sessions, she exceeded the criterion level after the 11th day. There was a significant increase in Zehra’s fluency performance in the intervention phase (average 16.6) compared to the baseline level (average 6.6).

Regarding the effect size, the computed effect size is as large as 1.00 for Doruk and Selin, and it is as large as 0.94 for Zehra. As seen in Fig. 1, the curves formed by the data obtained from the fluency performance measurements of all participants throughout the intervention gradually moved away from the baseline curves. At the same time, while the intervention continued with one participant, it was observed that the fluency performances remained at the same level in the probes made with the other participants. This shows that the change in the participant’s fluency performance resulted from the SP-PF training. In all participants, an immediate effect was observed with the start of the intervention. As a result, training with SP-PF was found to be effective in increasing the fluency level of all three participants in BAFs.

Maintenance data showed that fluency in BAFs performance was maintained at least at the fluency criterion level for all participants 7 and 15 days after the end of the intervention sessions. Doruk continued to maintain his fluency performance at least at the criterion level (even though it decreased slightly) 45 days after the intervention. Selin, on the other hand, could not maintain her fluency performance in the measurements 45 days later and regressed slightly (15 correct answers per minute). Zehra’s maintenance measurement, scheduled for 45 days later, could not be done because it coincided with school holidays.

Upon analysis of the data presented in Fig. 1, it is observed that the fluency levels of all students exhibited an upward trend when compared to their scores on the pretest. This indicates that the SP-PF intervention enabled students to achieve at least the criterion level when the fact and its addends were replaced.

### Social validity

A descriptive analysis of interview data with participants shows that, in general, all participants expressed positive opinions about the study. All three of the participants liked the application very much. They marked the statement indicating that their teachers and parents at school would appreciate their quick response to the addition facts. They also stated that they completely agreed with the idea that doing the addition facts so quickly would make it easier to perform more difficult facts. The results of the analysis of the answers given to open-ended questions are as follows: One participant mentioned the reinforcers as the feature she liked most about the study. The other one stated that it was incredibly good for her to learn to be faster at addition facts. One of them stated that he enjoyed working with the implementer very much and stated that he liked doing it without counting his fingers. All participants stated that there was no feature of the application that they did not like.

**Fig. 2:**
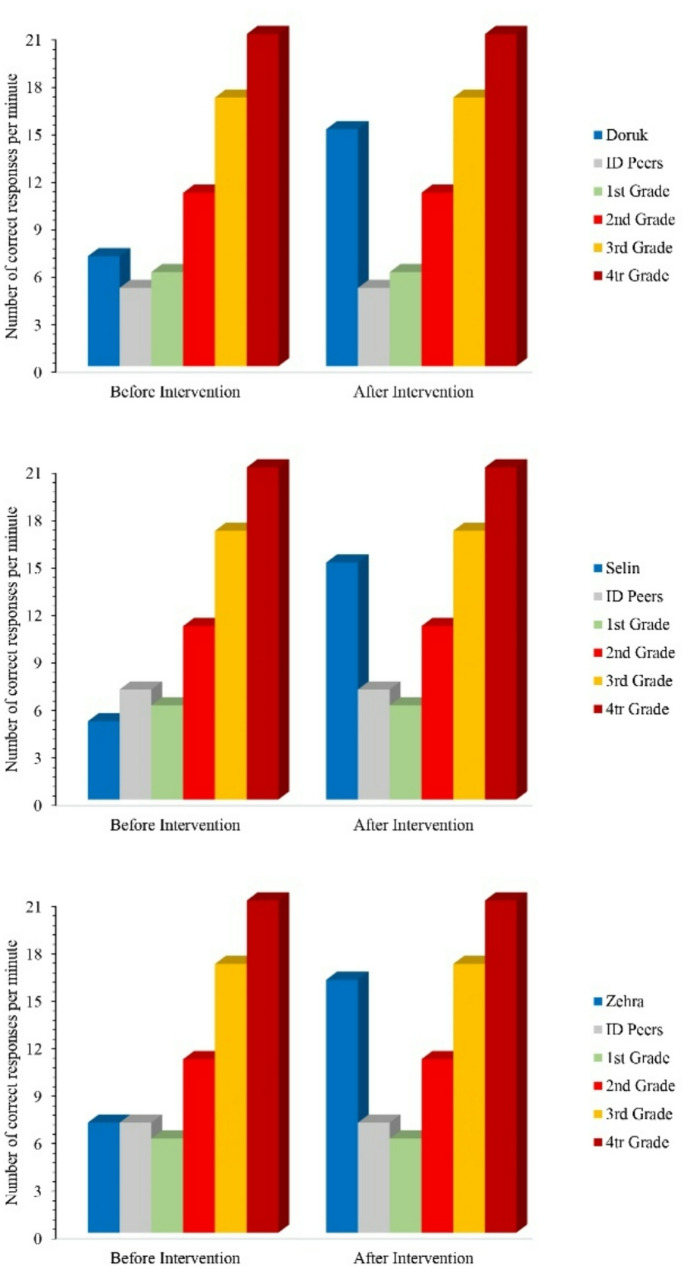
Social comparison findings of the participants’ level of fluency in BAFs

Social comparison findings regarding the fluency levels of the participants in BAFs are shown in Fig. 2. Doruk was slightly ahead of his peers with mild intellectual disabilities before the intervention. However, after the intervention, he increased the difference exponentially. Similarly, while he was slightly ahead of the 1st grade students with typical development at the beginning, he increased the difference after the intervention. Doruk was behind the 2nd grade students before the intervention, but after the intervention, he exceeded their average. When compared to the performance of 3rd and 4th grade students, Doruk was still behind after the intervention, but he mostly closed the gap. Figure 2 shows that while Selin was behind her peers with mild intellectual disabilities before the intervention, she performed well above the average of her peers after the intervention. Similarly, Selin was behind typically developing 1st and 2nd grade students before the intervention. After the intervention, she exceeded their averages. Selin was well behind the 3rd and 4th graders before the intervention. However, after the intervention, she came very close to the average of these two groups. As seen in Fig. 2, Zehra was performing equally with her peers with mild intellectual disabilities before the intervention. After the intervention, she exceeded the average of her peers. When compared to the performance of typically developing primary school students, Zehra outperformed 1st grade students before and after the intervention. However, at the end of the intervention, it was observed that she widened the gap. Zehra was behind the 2nd, 3rd, and 4th graders before the intervention. After the intervention, she surpassed the average of 2nd graders. Although she was still behind the average of 3rd and 4th graders, she closed the gap considerably.

## Discussion

This study aimed to examine the effects of SP-PF intervention on fluency in BAFs for students with mild intellectual disabilities. The results of this study indicate that SP-PF intervention was effective in improving fluency in BAFs for all participants. The level of fluency in BAFs of the participants improved at the criterion level. All the participants maintained their level of fluency in BAFs at least at the fluency criterion level up to 15 days and generalised across the features of change in BAFs. Effect size results in the study showed that the SP-PF procedure was highly effective for all participants, and visual analysis of the data also demonstrated these gains.

This study is a pioneering work among fluency in basic facts research that utilizes the SP-PF intervention, and it should be noted that no study has yet demonstrated the independent effects of SP and PF on fluency in BAFs. Further research is needed to analyze the separate contributions of these two components. Although a study specifically investigating the effect of SP on improving fluency in BAFs has not yet been conducted, two studies [17, 19] have demonstrated that interventions incorporating SP can positively influence fluency in multiplication facts. One such study examined the efficacy of SP on a student exhibiting low mathematics performance [19], while another investigated the impact of SP on two students with cognitive disabilities [17]. Given the dearth of studies examining the effectiveness of SP for students with special needs, this study contributes to the growing literature on SP by demonstrating its utility in promoting fluency in a variety of academic domains, beyond those initially explored in the existing research.

In this study, PF was implemented as a component of fluency in BAFs training. Although PF alone cannot be attributed to the effectiveness of the intervention, it is seen that interventions containing PF, like our study, have an influence on students’ performance [3, 27]. Gersten et al.‘s [20] meta-analysis study found that providing students with graphical information significantly increased their mathematics proficiency, while incorporating such elements into the intervention resulted in improved fluency.

Intervention packages that include different forms of PF (e.g., self-graphing) as an element of a self-management intervention are effective in increasing mathematics fluency [25, 26]. In these studies, participants exhibited improved overall academic and cognitive performance, and they themselves created graphical representations of their progress during instruction, which also included the application of cognitive strategies. Unlike these studies, we worked with students with mild intellectual disabilities, requiring systematic approaches. We advanced the existing studies by demonstrating the positive effect of PF with graphing by the implementer on teaching with SP. Of course, there is insufficient evidence to show whether the change in students’ math fluency performance in our study or in other interventions using PF was due to the strategy, PF, or the whole package. Codding et al. [24] found that there is no differentiation between treatments that cover-copy-compare, the combined all, and two types of PF with a bar graph (i.e., digits correct and incorrect per minute). They stated that due to the difficulty in determining whether the addition of PF produced better mathematics fluency for any of the participants. Future research is needed to compare interventions with and without PF to improve fluency in math.

In the study, all three students were able to maintain their fluency level at least until the 15th day. In the continuity measurements made with Doruk and Selin on the 45th day following the end of the training, their fluency performances tended to fall below the criterion level. Upon completing the training sessions, the teachers were informed about the level of fluency their students had achieved. They were provided with drill sheets and advised to make copies of these sheets for daily drills. However, these activities were not systematically monitored. During the visits for maintenance measurements, the teachers reported being unable to work on fluency regularly with the students. As Stein et al. [8] emphasize, intensive practice, systematic review, and sufficient time are essential components for effectively developing fluency in basic skills. Teachers should integrate routine, intensive, and cumulative fluency drills into their instruction for basic facts. As observed in this study, the lack of repetition and practice may have contributed to the slight decline in the 45th day measurements. The inability to organize a 45th day follow-up session with Zehra limits the discussion of this aspect. However, previous studies using SP have shown that students maintained their multiplication fluency performance for 42 days [17] and 45 days [19]. While our study’s findings are significant in demonstrating that two students achieved near criterion-level performance in the 45th day measurements, additional evidence is needed to draw more definitive conclusions on this matter.

Generalization was tested only in terms of stimulus generalization, where both the location of the facts and the addends were changed. Therefore, it cannot be claimed that generalization extends to other materials, environments, or individuals. The generalization findings of the study showed that three students with mild intellectual disabilities were able to perform at least at the criterion level. It should be noted that, unlike this study, other studies using SP measured generalization to different environments, individuals, and materials, with intervention packages that included flashcards [17] or systematic review and corrective feedback [19]. It is well-known that SP is an evidence-based teaching procedure [10], and this study has added this evidence to the literature that it is a procedure that serves generalization in fluency in facts. Differences in generalization between participants in this study may be related to individual characteristics or responses to the intervention. It is recommended to examine these factors in the future.

The social validity findings of the study indicate that the SP-PF intervention is effective and acceptable for students. It should be noted that the reference performance for social comparison is at the primary school level (grades 1–4), where fluency in BAFs is achieved. The comparison with peers with mild intellectual disabilities also shows that the students’ fluency performance increased rapidly and impressively (double that of their peers). In the study, for social validity, while the findings of social comparisons with both typically developing and intellectually disabled peers demonstrate the significant impact of the SP-PF procedure, it is important to note that the typically developing students were not matched by age or grade level but by their alignment with the primary school curriculum in terms of skill level. In other words, the comparison group consisted of primary school students who differed in age from the participants but were actively engaged in fluency studies. Additionally, it should be emphasized that the comparison with intellectually disabled peers who could perform addition fluently was based on a limited sample within the school attended by the participants.

At the end of the intervention phase, after observing significant effects in the three main participants, a study was conducted with the substitute participant (see Participants), not part of the main study, following ethical guidelines to apply effective interventions to substitute participants, or control groups. The substitute participant’s teacher received brief training on the SP-PF procedure and implemented it. During school visits for maintaining data collection from the main study participants, interviews revealed that the substitute participant’s fluency performance in BAFs increased from 3 cases per minute to 12. The teacher expressed surprise at the unexpected result, noting that such an outcome was typically not achievable. The teacher’s ability to quickly learn the process and the successful outcome of the intervention, despite the application not being as systematic as the experimental procedures, suggests that SP-PF could be a valuable procedure for both practitioners and researchers in the future.

In the study, several precautions were taken to minimize variables and ensure the research proceeded as planned, such as conducting one-on-one sessions with students in a separate room. However, it is important to note that SP-PF interventions can be easily implemented in various teaching environments without requiring a dedicated space, overly structured classes, or specialized materials. SP-PF can be applied in one-on-one or group settings within the classroom. Students may provide written or verbal responses, making fluency activities flexible and adaptable. SP is a simple and practical procedure. Short, frequent drills incorporating self-monitoring strategies like PF can effectively build fluency. As students monitor their own progress, they compete only with themselves, which supports individualized teaching, especially in inclusive settings.

Limitations of the study have consequences for future research, which will probably produce conclusions that help practitioners in their line of work. This study, which was shown to be effective for mild intellectual disabilities students who participated in the study, should be repeated with children with different characteristics, including typically developing children. The effect of conducting a study like this one with a group, which was conducted through 1–1 practitioner-student interaction, can be examined. Single-subject studies are frequently preferred research designs for demonstrating the effects of specific interventions, especially with special populations. However, the generalizability of findings in studies conducted with such small sample groups is limited. To overcome this limitation in the generalizability of results in single-subject studies, the same study should be repeated with students with similar characteristics. The number of BAFs was determined according to the targeted fluency criteria in line with the students’ own fluency performances, and all these facts (20 in total) were studied in each trial. Accordingly, the results of giving the facts in sets can be investigated. The social validity data for the application were not received from the teacher because the implementer was the researcher. In future studies, if teachers are practitioners, it can be measured how the intervention is evaluated in terms of real practitioners. Since SP and PF were used in combination in the study, it was difficult to predict, which caused the dramatic increase in students’ fluency performance. The effects of different training conditions can be compared: SP alone, PF alone, and SP and PF together.

Given the dearth of research on the efficacy of SP-PF intervention on fluency in basic facts among students with special needs, this study makes a significant contribution to the advancement of the literature on the use of SP and PF. While all three participants in the study were engaged in calculating their fingers to add numbers, by the end of the intervention, they demonstrated the ability to answer the facts presented without the use of their fingers. The students in question were identified by their teachers as being able to add two-digit numbers manually. Upon examination of the students’ mathematics notebooks, it was observed that they were studying addition by carrying. However, teachers expressed concern about the students’ tendency to move at a slow pace. Despite the students’ ability to add two-digit numbers, the absence of teacher assistance in enhancing their fluency indicates a significant issue. This study underscores this disparity, providing insight into the factors hindering fluency in students who can solve more difficult BAFs with fingers. It emerges as a critical point in understanding the broader implications of the study’s findings and the need for further investigation into why fluency enhancement is often overlooked in educational practices for students with special needs.

## Data Availability

All data generated and analysed during this study are included in this published article.

## Abbreviations

BAFs: Basic Addition Facts
IOA: Interobserver agreement
PF: Performance Feedback
SP: Simultaneous Prompting
SP-PF: Simultaneous Prompting with Performance Feedback
